# The Role of Obesity in the Regulation of Immunosuppressive Cell Infiltration and Immunosurveillance in Cancers

**DOI:** 10.3390/diseases13080271

**Published:** 2025-08-21

**Authors:** Chunye Zhang, Keyao Zhu, Jiazheng Liu, Ming Yang

**Affiliations:** 1 Bond Life Sciences Center, University of Missouri, Columbia, MO 65212, USA; 2 State Key Laboratory of Quality Research in Chinese Medicine, Macau University of Science and Technology, Taipa, Macau 999078, China; 3 Department of Surgery, University of Connecticut Health Center, Farmington, CT 06030, USA

**Keywords:** obesity, cancer, immune cell infiltration, immunosuppression, clinical trials, natural medicines, artificial intelligence

## Abstract

Cancer is a leading cause of death worldwide, causing about 10 million deaths annually. Obesity contributes to cancer progression by inducing chronic inflammation, immunosuppressive microenvironment, metabolic dysfunction, and therapeutic resistance. Accumulating evidence shows that obesity can advance the infiltration of immunosuppressive cells and ameliorate the function and cytotoxicity of tumor-killing cells such as natural killer cells, natural killer T cells, macrophages, and CD8 T cells in cancer patients, resulting in cancer progression. Understanding the molecular signaling pathways involved in obesity-induced immunosuppression and cancer cell proliferation enables us to screen new biomarkers for cancer early diagnosis and improve anti-tumor therapeutic efficacy in obese patients with cancer. In this review, we first review the molecular mechanisms by which obesity induces the immunosuppressive landscape in the tumor microenvironment and some key obesity-associated factors causing immunotherapeutic suppression and metabolic dysfunction. Then, the application of natural products in the treatment of obesity and obesity-associated cancers is summarized. In addition, we discuss the contradictory functions of obesity in cancer risk and treatment outcome. The potent roles of precision medicine and artificial intelligence in the management of obesity-related cancers are highlighted.

## 1. Introduction

In adults (aged ≥20 years), obesity is defined by a body mass index (BMI) ≥ 30 kg/m^2^. Among youth (aged 2 to 19 years), obesity is characterized by a BMI above the 95th percentile [1,2]. The World Obesity Federation has predicted that about 51% of the global population, or more than four million people, will be overweight or obese by 2035, which poses a huge economic impact [3]. Obesity is a major risk factor contributing to many chronic diseases, such as cardiovascular diseases (CVD) [4], metabolic dysfunction-associated steatotic liver disease (MASLD) [5], type 2 diabetes (T2D), and different types of cancers [6]. Many factors can cause obesity, including genetics, lifestyle, physical activity, aging, medical conditions, socioeconomic factors, and environmental factors [7,8]. Among these factors, excessive calorie intake and a sedentary lifestyle are the key drivers for obesity.

Cancer causes about 10 million deaths globally each year [9]. According to the American Cancer Society, the number of global cancer cases was predicted to increase to 35 million by 2025. The key risk factors leading to cancer include smoking, high BMI (overweight or obese), and infection [10]. Obesity can impact systemic alteration of metabolites, inflammation, hormone secretion, insulin sensitivity, and expression of cytokines and chemokines [11,12,13,14]. These factors can create a microenvironment to advance tumor initiation, progression, and invasion [15]. In addition, clinical studies show that obesity is closely associated with the development [16,17], prognosis [18,19], and treatment [20,21,22] of different types of cancers (Table 1). On the contrary, obesity has been reported to be negatively associated with the development of non-small cell lung cancer (NSCLC). The risk of NSCLC decreased in participants gradually across the BMI trajectory from a normal BMI at baseline to overweight or from overweight to obesity [23]. Another study also shows that obesity did not influence the management and treatment of breast cancer in the elderly population [24]. Thus, further investigation is required to test how obesity impacts tumor development and therapy.

To better understand the underlying mechanisms by which obesity can significantly impact cancer initiation and progression, we focus on investigating the role of obesity in regulating infiltration of immunosuppressive cells and immunosurveillance in cancer. The following aspects are discussed to elucidate how obesity contributes to cancer progression and resistance to immunotherapy, including obesity-induced chronic inflammation, immunosuppressive microenvironment, metabolic dysfunction, and immunotherapeutic resistance.

## 2. Obesity-Associated Factors in Inflammation, Energy Metabolism, and Cancer

Many different adipokines, cytokines, and chemokines are secreted by adipose tissues in obese subjects, which play an essential role in systemic inflammation and sugar and lipid metabolism. In this section, we review their roles in inflammation, energy metabolism, immune responses, and cancers.

### 2.1. Adipokines

Adipose tissue serves as a vital repository of fat. Excessive accumulation of adipose tissue causes obesity. Many adipokines, such as adiponectin and leptin, are secreted by adipocytes [25]. In addition to adipocytes, other cells in the adipose tissue, such as preadipocytes and infiltrating immune cells, can also contribute to the production of different adipokines and inflammation-associated cytokines [26]. These secreted factors can regulate insulin resistance, glucose and lipid metabolism, appetite, weight gain, energy intake and expenditure, inflammation, and immune responses [27,28]. Therefore, they play a significant role in health and diseases, including obesity-related cancers [29,30].

Adiponectin is mainly produced by fat cells to regulate glucose and lipid metabolism. It can enhance insulin sensitivity by binding to its receptors, such as adiponectin receptor 1 (AdipoR1) and AdipoR2, leading to the activation of downstream AMP-activated protein kinase (AMPK) and peroxisome proliferator-activated receptor alpha (PPARα) signaling pathways to maintain metabolic homeostasis [31]. However, patients with obesity have a decreased level of adiponectin, causing insulin resistance or reduced insulin sensitivity [32]. Adiponectin can impact cancer cell growth by regulating the cell cycle and apoptosis in different cancers [33], such as cervical cancer, breast cancer, and endometrial cancer. Therefore, it can also be applied to predict a better prognostic outcome in cancer patients [34].

Leptin secreted by adipose tissues can activate the signaling pathways of Janus kinase 2 (JAK2)/signal transducer and activator of transcription 3 (STAT3), extracellular signal-regulated kinases 1 and 2 (ERK1/2), c-Jun (transcription factor Jun), and AKT (protein kinase B) to promote tumor progression and migration [35,36]. For example, adipocyte-derived leptin and IL-6 can promote breast cancer cell metastasis by increasing the expression of lysyl hydroxylase (an enzyme in collagen synthesis) in tumor cells [37]. In obesity, overexpression of leptin increases cancer development risk, poor prognosis, and decreases the efficacy of immunotherapy against cancers [38,39]. The underlying mechanism is caused by the elevated inflammation and angiogenesis, cancer cell proliferation, and chemoresistance [40]. Although leptin plays an important role in inflammation and cancer development, it has a beneficial effect in enhancing the efficacy of cancer immunotherapy. Patients with obesity had better responses to immunotherapy in different cancers, such as melanoma and NSCLC, which was associated with an elevated leptin level [41,42,43]. This paradox highlights the complicated role of leptin in cancer therapy.

The level of visfatin is shown to be increased in obese subjects. Visfatin secreted from adipose tissues can activate phosphoinositide 3-kinase (PI3K)/AKT and ERK signaling pathways to promote tumor cell invasion and proliferation [44].

Resistin plays an important role in obesity-related carcinogenesis. It contributes to the growth and stemness of tumor cells in breast cancer through the signal transducer and activator of transcription 3 (STAT3) signaling pathway. Resistin can promote cancer cell proliferation through nuclear factor kappa B (NF-κB) and PI3K/AKT signaling pathways [45,46]. In addition, a high level of serum resistin has been reported to be correlated with poor overall survival of patients with breast cancer, which is caused by the activation of Toll-like receptor 4 (TLR4)/NF-κB/STAT3 signaling pathway [47,48]. Resistin is also considered as a prognostic marker for basal triple-negative breast cancer, which can induce tumor cell migration by activating the mitogen-activated protein kinase (MAPK) signaling pathway in breast cancer cells [49].

The expression of other adipokines, such as apelin and chemerin, is also elevated during obesity and has been reported to be associated with cancer cell survival, angiogenesis, and metastasis [50,51].

### 2.2. Cytokines

The proinflammatory cytokines secreted by adipose tissue-infiltrated macrophages and other immune cells, as well as the elevated production of free fatty acids (FFAs), can form a tumor cell-preferable microenvironment to increase the risk of cancer development and progression [52]. In obesity, adipose tissue chronic inflammation promotes the secretion of proinflammatory cytokines, such as tumor necrosis factor alpha (TNF-α), interleukin (IL)-1β, IL-6, IL-17, and interferon (IFN)-γ, which prolong the extension of chronic inflammation in adipose tissues [53]. Those secreted proinflammatory cytokines can promote cancer cell survival, proliferation, aggregation, and metastasis. An increased level of those cytokines has been detected to be associated with a poor prognostic outcome of patients with cancers and therapeutic resistance in cancer treatment [54,55]. Their involved signaling pathways contribute to both obesity and cancer. A study shows that an elevated level of T helper type 17 (Th17)-related cytokines, such as IL-17a, TNF-α, and IL-6, has a synergistic effect on the activation of STAT3/NF-kB signaling pathway, to promote colorectal cancer cell proliferation [56]. Upregulation of IL-1β has been found in both obesity and the tumor microenvironment. IL-1β stimulates the secretion of IL-6 by activating NF-kB signaling pathway, which promotes the progression and invasiveness of breast cancer [57]. Obesity aggregates tumor development and metastasis in ovarian cancer by upregulating IL-6 expression to boost the recruitment of myeloid-derived suppressor cells (MDSCs), causing overexpression of immune suppression-associated genes and cancer cell immune evasion [58].

An alteration of anti-inflammatory cytokines also occurs in both obesity and cancer. IL-15 plays an important role in the regulation of obesity-related metabolic diseases by inducing chronic adipose tissue inflammation [59]. IL-15 knockout mice have less diet-induced weight gain and accumulation of lipids in both visceral and subcutaneous tissues compared to control mice [59]. However, another study shows that a reduced expression of IL-15 is correlated with an increased body weight and adiposity in mice and humans [60]. Further studies also show that IL-15-mediated weight loss is independent of lymphocytes [60]. IL-15 confers anti-cancer function by stimulating immune response CD8^+^ T cells and natural killer (NK) cells against cancer cells. Thus, a decreased level of IL-15 in obesity may reduce the anti-tumor immune response. An upregulation of adipose tissue-associated IL-2 expression in overweight or obese individuals contributes to an increased expression of metabolic parameters, such as C-reactive protein and triglyceride levels, which increases the risk of the development of insulin resistance. Adipose tissue-associated IL-2 is also associated with overexpression of a variety of inflammatory markers, such as IL-8, IL-12a, IL-1β, C-C motif chemokine ligand 5 (CCL5), CCL15, TLR2, and an inflammatory macrophage marker (CD11c), inducing inflammation and insulin resistance [61].

### 2.3. Chemokines

Chemokines play a pivotal role in the recruitment or attraction of immune cells, such as monocytes and macrophages, which leads to the augmentation of inflammation and immune cell infiltration in adipose tissues [62,63]. Similarly, in the tumor microenvironment, chemokines contribute to the alteration of immune cell profiles by regulating immune cell infiltration [64]. Moreover, chemokines are responsible for promoting tumor angiogenesis, tumor cell invasion, and migration. Adipose tissue-derived CCL5 can aggravate the immune response of proinflammatory monocytic myeloid-derived suppressor cells (M-MDSCs) (CD11b^+^Ly6G^−^Ly6C^hi^) and promote proinflammatory M1 macrophage polarization. Therefore, CCL5 can exacerbate tissue inflammation and decrease insulin sensitivity in obese mice [65]. Cancer-associated fibroblasts-derived CCL5 is responsible for the progression of hepatocellular carcinoma (HCC) and cancer cell metastasis. The cancer cell metastasis is mediated by hypoxia-inducible factor 1α (HIF1α)/zinc finger E-box binding homeobox 1 (ZEB1) axis, which is involved in the process of epithelial–mesenchymal transition (EMT) [66]. In triple-negative breast cancer, the CCL5-CCR5 axis plays an essential role in the infiltration of immunosuppressive myeloid cells and neutrophils. Bone marrow-derived CCL5 regulates tumor-associated macrophage differentiation to promote tumorigenesis [67]. In an obese mouse model of pancreatic cancer, depletion of tumor-derived C-X-C motif chemokine ligand 5 (CXCL5) enhances the efficacy of anti-programmed cell death protein 1 (PD-1) immunotherapy, which is mediated by the upregulation of CD8^+^ T cell tumor infiltration [68]. CXCL8 derived from cancer-associated adipocytes shows a synergistic effect on anti-tumor immune response by inducing CD4^+^ T cell and CD8^+^ T cell infiltration and upregulating CD274 expression [69].

Other chemokines include but not limited to CCL2 (MCP-1), CCL3 (MIP-1α), CCL4 (MIP-1β), CCL19, CXCL1, CXCL8 or IL-8, CXCL10, and CXCL12 also play an important role in obesity and cancer immunotherapy [70,71]. In summary, obesity-associated changes in chemokine expression can regulate inflammation and immune responses to influence cancer initiation and progression.

## 3. Obesity-Associated Immunosuppressive Microenvironment

Obesity can induce an immunosuppressive microenvironment in the tumor. A recent study demonstrates that obesity can induce the expression of PD-1 on tumor-associated macrophages, which is mediated by the activation of mechanistic target of rapamycin (mTOR) and MYC (MYC proto-oncogene, bHLH transcription factor). This process causes the dysfunction of microphages, featured by an increased level of oxidative phosphorylation, elevated mitochondrial mass, impaired phagocytosis, and a decreased expression level of major histocompatibility complex (MHC)-II [72]. Therefore, obesity-associated alteration of PD-1 expression and macrophage dysfunction suppresses anti-tumor immune response or induces an immunosuppressive microenvironment. In contrast, treatment targeting PD-1 expression on macrophages can enhance the anti-tumor function of T cells [72]. Another study shows that high-fat diet-induced obesity can promote cancer growth in a mouse model of breast cancer induced by the implantation of cancer cell line E0771. Obesity weakened anti-tumor immune responses by reducing the infiltration of CD8^+^ T cells and the ratio of M1/M2 macrophages.

The recruitment and infiltration of immunosuppressive cells dysregulate the phenotypes and cytotoxicity of tumor-killing cells, such as NK cells, natural killer T (NKT) cells, macrophages, and CD8^+^ T cells in many cancers, which advances cancer progression. For example, in obese subjects with breast cancer, the level of regulatory T cells (Tregs) in visceral adipose tissue is increased, which causes tumor cell proliferation [73]. These studies highlight the importance of immune profile alteration in obesity and its impact on tumor growth. In this section, several examples are discussed to show how obesity can regulate the anti-tumor immune response in the tumor microenvironment.

### 3.1. CD8 T Cells

**Figure 1:**
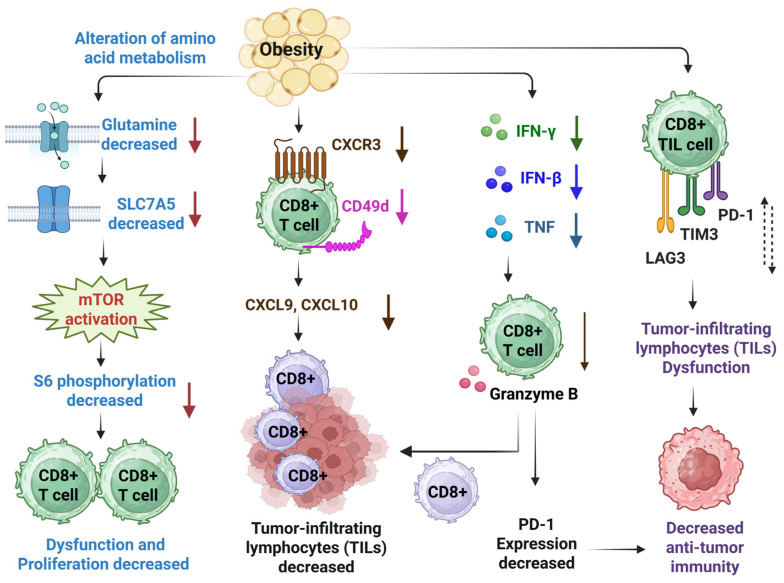
Obesity induces an immunosuppressive microenvironment by regulating CD8^+^ T cell function. Obesity induces the alteration of amino acid metabolism, resulting in a decreased expression level of glutamine and solute carrier family 7 member 5 (SLC7A5). Consequently, it triggers the activation of the mTOR signaling pathway and leads to a decreased proliferation of CD8^+^ T cells. It also causes the dysfunction of CD8^+^ T cells. Obesity induces decreased levels of tumor-infiltrating lymphocytes (TILs) mediated by downregulating CXCR3, CD49d, CXCL9, and CXCL10 expression. Obesity reduces the secretion of IFN-γ, IFN-β, and TNF, resulting in less production of granzyme B. This process contributes to the compromised level of TILs and decreased PD-1 expression. Therefore, the anti-tumor immunity of CD8^+^ T cells is impaired. Obesity causes reduced expression of PD-1, Tim3, and Lag3 on CD8^+^ T cells in melanoma cancer, weakening anti-tumor immunity. The cartoons in this figure were prepared using Biorender (https://biorender.com).

Obesity alters amino acid metabolism, resulting in a decreased uptake of glutamine. A study reveals that a reduced level of glutamine can impact the activity of solute carrier family 7 member 5 (SLC7A5) and phosphorylation of S6 (known as mTOR downstream signaling) [74]. This alteration decreases CD8^+^ T cell proliferation and induces CD8^+^ T cell dysfunction (Figure 1). Obesity-induced metabolic alteration also causes reduced expression of Ki-67 (antigen Kiel 67), IFN-γ, and phosphorylation of S6 (pS6), resulting in a decreased level of kynurenine uptake and a reduced CD8^+^ T cell activation, consequently inducing an immunosuppressive condition. In addition to the decreased level of CD8^+^ T cells, obesity also induces decreased expression levels of CXCR3, CD49d, CXCL9, and CXCL10. The reduced expression of CXCL9/10-CXCR3 ameliorates the number of tumor-infiltrating lymphocytes (TILs). Moreover, obesity suppresses the expression of IFN-γ, IFN-β, TNF, and granzyme B (GzmB) in CD8 T cells. Another study also reveals that obesity induces a decreased expression level of PD-1, lymphocyte-activation gene 3 (Lag3), and T cell immunoglobulin and mucin domain-3 (Tim3) in tumor-infiltrating CD8^+^ T cells, resulting in CD8^+^ T cell dysfunction [75].

However, clinical studies show that obesity has divergent effects in cancer immunotherapy. The contradictory role of obesity in cancer risk and immunotherapy outcome is shown in patients with renal cancer [76]. For example, obese patients with renal cell carcinoma (RCC) had shorter progression-free survival (PFS) with anti-PD-1 therapy compared to non-obese patients with RCC. The underlying mechanism is associated with the levels of immune infiltration of PD-1^high^CD8 T cells and proinflammatory cytokine IL-1β [77]. In contrast, other studies find that overweight or obese RCC patients had longer PFS, better overall survival outcomes, and lower time-to-treatment failure after receiving anti-PD-1/PD-L1 treatments compared to control non-obese groups [78]. Thus, the role of obesity in impacting anti-tumor immunotherapy outcome is complex, and the underlying biological mechanisms need to be further investigated.

Moreover, for overweight and obese individuals with cancer, many confounders are potential contributors that influence the immune landscape and the subsequent therapeutic outcomes. For instance, the accuracy of adjusting dosages (commonly based on body weight) of chemotherapeutic drugs could be one of the challenges. In such conditions, many factors need to be taken into consideration, such as cytotoxicity and drug absorption, distribution, metabolism, and excretion. The interplay of molecular and signaling pathways between obesity and cancer can also impact the therapeutic efficacy of drugs. However, to what extent of the dosage is calculated based on bodyweight in obese individuals might influence the drug treatment outcome is unclear [79]. The impact of obesity on cancer therapy and immune cell function in the tumor microenvironment needs to be further investigated.

### 3.2. Macrophages, MDSCs, and NKT Cells

**Figure 2:**
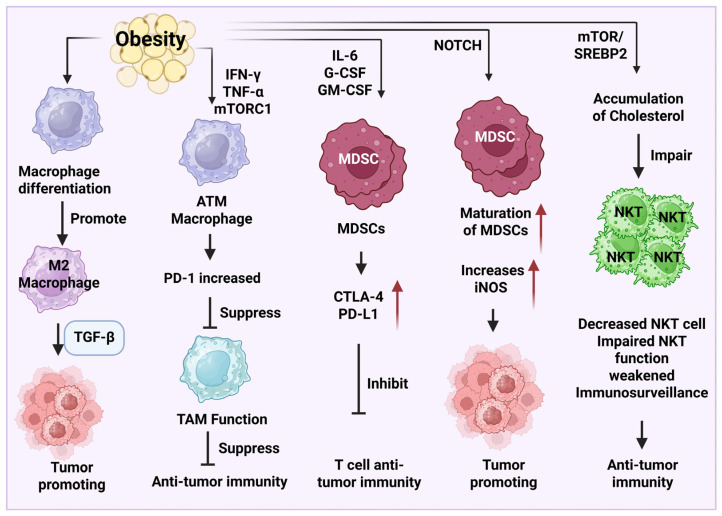
Obesity induces an immunosuppressive microenvironment by regulating the infiltration and function of macrophages, MDSC, and NKT cells. Obesity promotes tumor growth and proliferation by regulating the expression of transforming growth factor-β (TGF-β) and M2 macrophage polarization. Obesity promotes elevated expressions of cytotoxic T-lymphocyte-associated protein 4 (CTLA-4) and programmed death-ligand 1 (PD-L1) through the recruitment of myeloid-derived suppressor cells (MDSCs), suppressing T cell anti-tumor immunity. Activation of the NOTCH signaling pathway in obesity promotes the maturation of MDSCs and the upregulation of inducible nitric oxide synthase (iNOS), contributing to tumor progression. The activation of mechanistic target of rapamycin (mTOR)/sterol regulatory element-binding protein 2 (SREBP2) signaling induces the accumulation of cholesterol, resulting in a low level of NKT cell number and immunosurveillance dysfunction. Consequently, it weakens the anti-tumor immunity of NKT cells. The cartoons in this figure were prepared using Biorender (https://biorender.com).

The expression of transforming growth factor-β (TGF-β) in M2-like macrophages during obesity can promote tumor cell proliferation and induce an immunosuppressive microenvironment (Figure 2). One study demonstrates that adipose tissue macrophages (ATMs) have elevated expression levels of PD-1, which is mediated by the production of IFN-γ, TNF-α, and mTORC1. However, PD-1 expression can decrease the functionality of ATMs to suppress their anti-tumor function [80]. A novel macrophage subtype, lipid-associated macrophages (LAMs), also known as CD9^+^ macrophages, has been identified to play an important role in tissue metabolic homeostasis. The expansion of LAMs is found in the adipose tissues in obese mouse models, which confers an anti-inflammatory effect. ATMs maintain tissue homeostasis by reducing serum insulin, blood cholesterol levels, and glucose intolerance. The underlying mechanism of LAM function is driven by a lipid receptor, Trem2 (triggering receptor expressed on myeloid cells 2) [81].

Obesity can also promote tumor growth and inhibit T cell antitumor function through the modulation of MDSCs. Obesity induces elevated expression levels of IL-6, granulocyte colony-stimulating factor (G-CSF), and granulocyte-macrophage colony-stimulating factor, which increase the expression of cytotoxic T-lymphocyte-associated protein 4 (CTLA-4) and programmed death-ligand 1 (PD-L1) to suppress T cell cytotoxicity [82]. Additionally, obesity promotes the maturation of MDSCs and increases the expression of inducible nitric oxide synthase (iNOS) through neurogenic locus notch homolog protein (NOTCH) signaling pathway (Figure 2), leading to a tumor-promoting effect [83]. Using a diet-induced obesity mouse model with breast cancer, researchers demonstrate that an elevated expression level of intratumor CXCL1 can induce the accumulation of CXCR2-expressing G-MDSC into the tumor microenvironment. However, immunosuppressive cell infiltration causes CD8^+^ T cell apoptosis and immunotherapy resistance [84].

In a diet and chemical (azoxymethane)-induced obesity-associated colon cancer model, NK cell number and functions are decreased when compared to NK cell number and functions in the non-obese tumor model. Meanwhile, the severity of tumor progression is also higher in the obese group compared to that in the normal weight group [85]. Obese individuals have fewer circulating NK cells and decreased NK cell functions, including decreased cytotoxicity, reduced secretion of perforin and granzyme, and weakened metabolic activity. The dysfunction of NK cells increases the proliferation of malignant tumor cells and promotes tumor development [86,87,88].

Obesity reshapes the metabolic profile in the tumor microenvironment to induce tumor immunosuppression. For instance, a study reveals that the accumulation of cholesterol in obesity can reduce NKT cell number and impair their function to reduce their immunosurveillance in HCC [89]. Moreover, obesity can regulate the mutation of oncogenes, such as Kirsten rat sarcoma virus (KRAS), epidermal growth factor receptor (EGFR), SET domain-containing 2 protein (SETD2), and BRCA1-associated protein 1 (BAP1), which also increases the risk of cancer development [90].

Obesity-associated gut dysbiosis can disrupt gut homeostasis and induce an imbalance of gut microbial species, which might result in an expansion of tumor-promoting pathogenic bacteria and a decrease in tumor-preventive bacteria. For example, treatment with a high-fat diet can increase colorectal tumorigenesis in mice by increasing levels of tumor-promoting bacteria *Alistipes* sp. Marseille-P5997 and *Alistipes* sp. 5CPEGH and a decreased amount of probiotic strain *Parabacteroides distasonis* [91]. In addition, gut microbiota dysbiosis can damage the tight junctions of epithelial cells in the gut, causing leakage of microbial components and metabolites into the circulating system and systemic inflammation. It also induces abnormal metabolism and dysregulation of energy metabolism to cause immune evasion and tumorigenesis in cancers, such as breast cancer [92].

Obesity regulates breast cancer progression and therapy. In mice with obesity-related breast cancer, anti-PD-1 treatment significantly suppressed tumor progression by increasing macrophage M1 polarization and the populations of dendritic cells and cytotoxic CD8 T cells. The alteration of immune cell profiles was also shown in breast cancer patients with anti-PD-1 therapy. Additionally, the abundances of some gut microbiota, such as *Bifidobacterium* and *Lactobacillus*, were closely associated with the efficacy of immune checkpoint blockade (ICB) therapy [93]. In obese mice with breast cancer, anti-PD-1 treatment uniquely increased the abundances of gut microbiota [93], such as *Odoribacter*, *Adlercreutzia*, and *Mogibacteriaceae*.

A meta-analysis study was performed to dissect the role of obesity or BMI on the survival outcomes of NSCLC patients with the treatment of immune checkpoint inhibitors. The results revealed that overweight and obese patients had prolonged or increased overall survival (OS) and PFS compared to patients with normal weight. It is commonly known that overweight and obesity can increase the risk of cancer development; however, obese patients had better survival outcomes compared to lean individuals in this meta-analysis study [94]. It is known as a phenomenon of obesity paradox. The results suggest there are complex roles of obesity in some cancers.

## 4. Important Signaling Pathways Involved in Obesity-Related Cancer Development and Therapy

There are some key signaling pathways involved in obesity-related cancer development and immunosuppression. Here, we list some examples to discuss their functions.

### 4.1. JNK, IKK/NF-κB, and STAT3 Signaling Pathways

An elevated level of FFAs in obesity can activate some inflammatory kinases, such as c-Jun N-terminal kinases (JNK), inhibitory-κB kinase (IKK)/NF-κB, and STAT3 in liver and adipose tissues. Their activation can induce insulin resistance, inflammation, tumor cell proliferation and metastasis, and immune suppression [95,96,97]. The activation of JNK expression in macrophages is necessary for M1 macrophage polarization (proinflammatory phenotype) and in the process of obesity-induced insulin resistance [98,99]. Activation of JNK promotes the serine phosphorylation of insulin receptor substrate 1 (IRS-1), which disrupts the insulin receptor signaling pathway to influence glucose uptake, eventually resulting in insulin resistance [100].

The IKK/NF-κB signaling pathway can be activated to increase the production of cytokines (e.g., TNF-α, IL-6, and IL-1β) to promote inflammation and insulin resistance. The activation of STAT3 stimulates the secretion of IL-6 and activates JNK, mTOR, and protein kinase C (PKC) signaling pathways, which aggravate insulin resistance [101].

In cancer, JNK is well known for its role in regulating tumorigenesis. The activation of JNK is inhibited by NF-κB expression, resulting in cancer cell survival. The JNK signaling pathway also promotes an immunosuppressive tumor microenvironment in triple-negative breast cancer. Activation of IKK/NF-κB promotes the release of proinflammatory cytokines to aggravate EMT, angiogenesis, and immune suppression in tumors [102]. Activation of STAT3 and NF-κB can directly promote tumor cell survival, proliferation, metastasis, EMT, angiogenesis, and immune suppression. Moreover, obesity-induced expression of lipocalin 2 also contributes to the activation of NF-κB and STAT3 to facilitate M1 macrophage polarization, exacerbating the insulin resistance and inflammation [103].

### 4.2. PI3K/AKT/mTOR Signaling Pathway

**Figure 3:**
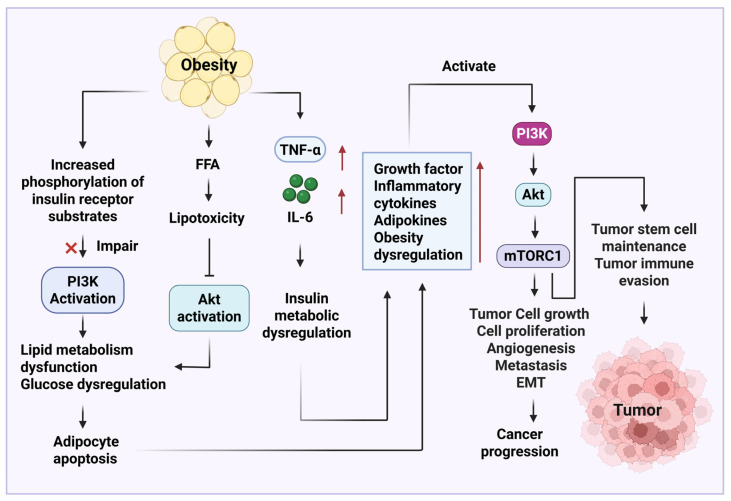
Obesity promotes tumor growth through the activation of phosphoinositide 3-kinase (PI3K)/protein kinase B(AKT)/mammalian target of rapamycin (mTOR) signaling pathway. Obesity-induced deactivation of PI3K and activation of the AKT signaling pathway contribute to adipocyte apoptosis. Obesity-induced secretion of inflammatory cytokines, such as IL-6 and TNF-α, promotes insulin metabolic dysregulation. Both adipocyte apoptosis and insulin metabolic dysfunction further exacerbate the elevated levels of growth factor, inflammation, and adipokines, resulting in the activation of PI3K/AKT/mTOR to cause cancer progression. The cartoons in this figure were prepared using Biorender (https://biorender.com).

Obesity can increase serine phosphorylation of insulin receptor substrates to reduce PI3K activation, which results in a dysregulation of glucose and lipid metabolism [104]. This process led to adipocyte apoptosis. Additionally, obesity induces an increased expression of inflammatory cytokines, such as IL-6 and TNF-α, to promote insulin resistance and metabolic dysfunction [105]. Obesity-induced elevated levels of FFAs can aggravate lipotoxicity, consequently inhibiting the activation of AKT downstream signaling pathways [106]. This process induces glucose and lipid metabolism dysregulation and adipocyte apoptosis. The activation of PI3K/AKT/mTOR signaling pathway in obesity induces hepatic steatosis and accumulation of visceral fat, further promoting liver and systemic inflammation [107]. In cancer, obesity-induced expression of growth factors, inflammatory cytokines (e.g., IL-6 and TNF-α), and adipokines (e.g., leptin) can induce dysregulation PI3K/AKT/mTOR signaling pathway, to induce cancer progression and predict a poor prognosis. Alteration of the PI3K/AKT/mTOR signaling pathway also contributes to tumor cell survival, proliferation, angiogenesis, metastasis, EMT, and chemotherapy resistance (Figure 3). In addition, the PI3K/AKT/mTOR signaling pathway is critical for maintaining the survival of tumor stem cells and causing tumor evasion [108,109]. Finally, obesity-induced hyperinsulinemia and inflammation impact the PI3K-targeted immune therapy [110].

### 4.3. Wnt/β-Catenin Signaling Pathway

**Figure 4:**
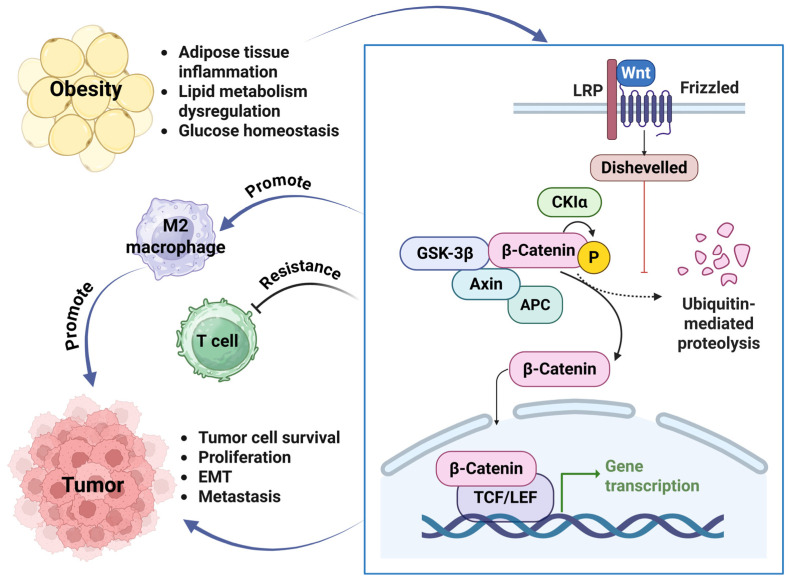
Obesity promotes tumor growth through activation of the Wnt/β-catenin signaling pathway. Obesity-induced inflammation and disruption of lipid metabolism and glucose homeostasis activate the Wnt signaling pathway, causing the failure of ubiquitin-mediated proteolysis of β-catenin. This β-catenin could then be involved in promoting the gene transcription processes. This process promotes tumor cell survival, cell propagation, EMT, and metastasis. The activation of the Wnt/β-catenin signaling pathway also promotes M2 macrophage polarization and resistance to T cell cytotoxicity. The cartoons in this figure were prepared using Biorender (https://biorender.com).

In obesity, the Wnt/β-catenin signaling pathway (Figure 4) plays a key role in promoting liver steatosis, adipose tissue inflammation, and dysregulation of lipid metabolism and glucose homeostasis [111,112,113]. In cancers, it also contributes to liver steatosis and inhibition of adipogenesis. Activation of Wnt/β-catenin promotes the M2 macrophage polarization to enhance tumor cell propagation and resistance to T cell cytotoxicity [114,115]. In NSCLC, the Wnt/β-catenin signaling pathway is responsible for immune escape and resistance to immunotherapies, such as anti-PD-1/PD-L1 antibody and anti-CTLA-4 antibody [116]. Wnt/β-catenin can also regulate dendritic cell recruitment in a murine melanoma model by inducing the activation of transcription factor 3 to suppress CCL4 expression [117].

### 4.4. NOTCH Signaling Pathway

In obesity, the NOTCH signaling pathway is activated in preadipocytes, which is responsible for weight gain and disruption of glucose homeostasis, and decreases insulin sensitivity in white adipose tissues. The elevated level of FFAs in liver adipose tissues during obesity can activate the NOTCH signaling pathway, to increase insulin resistance (IR), production of reactive oxygen species (ROS), and lipophagy. NOTCH promotes mTORC1 activation to subsequently increase the production of triglycerides (TGs), causing an increase in the production of VLDL and lipophagy [118].

In cancer, activation of the NOTCH signaling pathway increases the production of cytokine IL-1β and chemokine CCL2, which induce inflammation and recruitment of tumor-associated macrophages. The NOTCH signaling pathway also modulates TGF-β and CXCL5 secretion to promote the infiltration of tumor-associated neutrophils, and reprograms metabolic processes, such as switching glycolytic processes, to induce tumor growth. The NOTCH signaling pathway is also involved in tumor stem cell renewal, EMT, and tumor angiogenesis [119].

NOTCH signaling pathway mediates M1 and M2 polarization of tumor-associated macrophages to display anti-tumor and tumor-promoting functions, respectively [120]. NOTCH can increase anti-tumor immune response by regulating CD8^+^ effector T cells, and cause tumor-promoting function by modulating PD-1-induced exhaustion of CD8^+^ T cells [121]. This pathway impacts CD4^+^ T cell function and differentiation in cancers. Type 1 T helper cell (Th1) plays an anti-tumor role, whereas Th2 and regulatory T cells (Treg) play the function of tumor-promoting immunity [122].

### 4.5. HIF-1α Signaling Pathway

**Figure 5:**
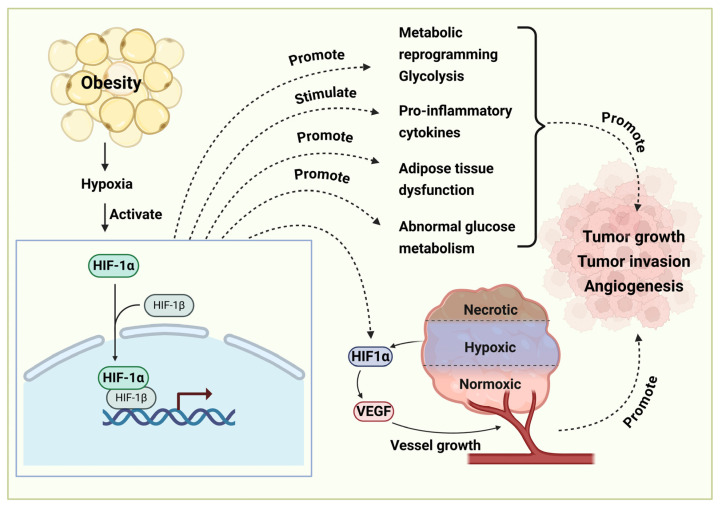
Obesity promotes tumor growth through the activation of the HIF-1α signaling pathway. Obesity-induced hypoxia can activate the expression of hypoxia-inducible factor 1 alpha (HIF-1α) to promote gene transcription. Subsequently, it promotes tumor development via reprogramming metabolic processes (e.g., increasing glycolysis), inflammation, adipose tissue dysfunction, and abnormal glucose metabolism. Activation of the HIF-1α signaling pathway promotes tumor vessel growth by upregulating vascular endothelial growth factor expression. The cartoons in this figure were prepared using Biorender (https://biorender.com).

Obesity-induced hypoxia in adipose tissues activates the expression of hypoxia-inducible factor 1 alpha (HIF-1α), which can stimulate the release of proinflammatory cytokines to induce inflammation (Figure 5). The activation of HIF-1α impairs adipocyte function and metabolism, causing an energy metabolism imbalance and insulin resistance [123]. The activation of HIF-1α also promotes angiogenesis to promote cancer cell survival, proliferation, and metastasis [124,125]. HIF-1α activation is also involved in metabolic reprogramming, such as increasing glycolysis, which promotes tumor invasion and angiogenesis [126].

Obesity-associated signaling pathways, such as Wnt/β-catenin, PI3K/AKT/mTOR, NOTCH, and JAK/STAT3 signaling pathways, are involved in immunotherapy resistance. In addition, they are implicated in the maintenance and survival of cancer stem cells. Targeting cancer stem cells can be a promising strategy to cope with immunotherapy resistance and manage cancer relapses post-immunotherapy. Integration of multiple approaches in investigating obesity-associated cancers is beneficial to facilitate the identification of novel biomarkers and therapeutic targets for cancer treatment [127]. In addition, spatial technologies and multi-omics such as transcriptomics, metabolomics, proteomics, genomics, and epigenomics can advance our understanding of obesity-related factors in cancer development and therapy [128].

## 5. Natural Products in Obesity-Associated Cancer Therapy

As a chronic disease caused by multiple factors, obesity predisposes individuals to various cancers. Natural medicines such as phytomedicines demonstrate irreplaceable advantages in the treatment of obesity and cancer due to their multi-component and multi-target characteristics. These medicines can not only reshape anti-tumor immune responses by ameliorating or reducing immunosuppression, metabolic disorders, and secretion of obesity-associated inflammatory factors in the tumor microenvironment, but also can break the malignant progression chain from obesity to cancer via regulating the balance of the immune system and energy metabolism. The findings in the functions of natural products provide new therapeutic strategies for tumor prevention and treatment, and open new avenues for deciphering the mechanisms of developing multi-target therapies in metabolic disorders, including cancers.

### 5.1. Ginsenosides

Ginsenosides, a complex of steroid saponins, are extracted from ginseng, the root of the plant *Panax*. Ginseng extract can treat obesity-associated type 2 diabetes [129]. Many experiments have demonstrated that treatment with ginseng extract induces weight loss in mice, rats, and humans [130,131,132,133,134,135,136]. Ginsenoside Rg3, an active component of ginsenosides, can inhibit oxidative stress and the formation of advanced glycation end products (AGEs) [137]. Amino acid derivatives of ginsenosides can also suppress the digestion and absorption of carbohydrates in the gastrointestinal tract, thereby reducing blood sugar levels [138]. Furthermore, ginsenosides can enhance the binding ability and cytotoxicity of NK cells to tumor cells, thus displaying the potential in treating diseases driven by NK cell dysfunction and chronic inflammation. For example, ginsenoside Rh2 inhibited the growth and metastasis of postmenopausal breast cancer cells [139].

### 5.2. Artemisinin

Artemisinin, extracted from *Artemisia annua* (sweet wormwood), has garnered significant attention for its role in the treatment of obesity and obesity-related metabolic diseases. In obese individuals, the expression of inflammatory cytokine TNF-α is elevated, which is a key marker for systemic inflammation. Artemisinin exerts an immunosuppressive function by suppressing B cell and pathogenic T cell activation and increasing regulatory T cell expansion [140]. Artemisinin and its derivatives can also suppress endoplasmic reticulum stress to prevent obesity progression [140]. They can upregulate the expression levels of uncoupling protein 1 (UCP1), peroxisome proliferator-activated receptor gamma coactivator 1-α (PGC1α), and PR domain containing 16 (PRDM16), to promote adipocyte browning or enhance UCP1 expression in the inguinal adipose tissues of C57BL/6J mice to increase thermogenesis, thereby reducing bodyweight [141].

Furthermore, the anti-cancer mechanism of artemisinin is closely related to its molecular properties. The endoperoxide bridge in its structure interacts with intracellular iron ions or heme groups, generating cytotoxic effects [142]. Increased intracellular irons in tumor cells can significantly boost the cytotoxicity of artemisinin [143]. Due to their increased demand for iron, cancer cells exhibit higher rates of iron metabolism and increase expression levels of transferrin receptors compared to normal cells [144], which increases the cytotoxicity of artemisinin to tumor cells.

### 5.3. Paclitaxel

Paclitaxel is an active monomer applied to treat various cancers. For example, it can inhibit the proliferation of endometrial cancer (EC) cell lines, induce EC cells to undergo apoptosis, enhance cellular stress responses, and cause cell cycle arrest in the G1 phase [145]. Obesity is a high-risk factor causing the development of EC. Studies show that paclitaxel combined with weight loss management (e.g., intermittent energy restriction and administration of low-fat diets) can reverse the obesity-induced abnormal elevation of serum insulin, leptin, and inflammatory factors, and reduce tumor incidence and weight, thereby reversing the cancer-promoting effect of obesity in mouse EC models [146].

Obesity is a contributing factor in the development and diagnosis of ovarian cancer [147]. Epithelial ovarian cancer (EOC) is the gynecological malignancy with the highest mortality rate, and the primary target organ for local metastasis is the omentum, which is rich in adipocytes. Research has found that adipocytes and omentum-derived stromal cells can enhance ovarian cancer resistance to paclitaxel [148], a mechanism related to fatty acid metabolism-associated stemness. Omental adipocytes can drive the EMT of EOC cells to promote their invasion and resistance to chemotherapy [149].

### 5.4. Hesperidin

Hesperidin is a flavanone glycoside extracted from citrus peels, and it possesses anti-obesity and anti-cancer activities by reducing cholesterol levels and blood pressure [150]. Regarding its anti-obesity functions, hesperidin exhibits multi-dimensional therapeutic properties. It can stimulate the release of appetite-regulating cholecystokinin (CCK) from intestinal endocrine STC-1 cells, thereby exerting an anti-obesity effect through appetite suppression [151]. It also inhibits the expression of several key targets involved in adipogenesis, including CCAAT enhancer-binding protein β (C/EBPβ), sterol regulatory element binding protein 1c (SREBP1c), peroxisome proliferator-activated receptor γ (PPAR-γ), and perilipin [152]. In addition, hesperidin can suppress fat accumulation by inhibiting the expression of stearoyl-CoA desaturase [153].

Hesperidin can enhance the production of ROS in tumor cells. It induces apoptosis by upregulating caspase family members and activating the mitochondrial apoptosis pathway [154] and inhibiting the cell proliferation marker [155]. Hesperidin can also arrest the cancer cell cycle in the G0/G1 and G2/M phases and regulate angiogenesis to promote cancer growth [156].

### 5.5. Quercetin

Quercetin is a flavonol found in many plants. In improving obesity and related metabolic syndrome, it can promote adiponectin secretion from adipocytes via a PPAR-independent pathway [157]. Furthermore, oral administration of quercetin can enrich intestinal *Lactobacillus*, modulate gut microbiota structure, to consequently promote the production of non-12α-hydroxylated bile acids in serum. In brown fat and browning of white fat, bile acids can interact with their receptor Takeda G protein-coupled receptor 5 (TGR5) to increase energy metabolism, thereby alleviating metabolic dysfunction in obese models [158].

In direct anti-cancer action, quercetin exerts inhibitory effects through multiple targets [159]. It can cause cell cycle arrest by several mechanisms, such as inhibiting the promoter activity of cyclin B1, suppressing the MAPK/ERK1/2 signaling pathway, and activating the transcription factor p53. Simultaneously, quercetin promotes apoptosis through mechanisms, including activation of TGF-β (transforming growth factor-beta) signaling pathway, and inhibition of pro-survival signaling pathways like PI3K/AKT/mTOR, Wnt/β-catenin, NOTCH, sonic hedgehog protein (SHH), and JAK/STAT, thereby impeding DNA repair [160]. Additionally, quercetin can inhibit tumor angiogenesis by regulating the vascular endothelial growth factor (VEGF) and its receptor axis to stimulate tumor blood vessel formation, consequently inhibiting tumorigenesis and development [160].

### 5.6. Celastrol

Celastrol, a natural active ingredient extracted from the root bark of Tripterygium wilfordii, possesses multi-dimensional anti-obesity effects by acting on lipid metabolism, inflammatory responses, energy metabolism, and gut microbiota modulation. Specifically, it can reduce macrophage infiltration and inflammatory cytokine production in the liver and adipose tissues, alleviating metabolic disorders in obese mice by inhibiting TLR3/NLRP3 (NLR family pyrin domain containing 3) inflammasome activation [161]. Additionally, it can directly induce cell apoptosis of preadipocytes by interacting with RAB7 (Ras-related protein 7) and VAMP7 (vesicle-associated membrane protein 7) to inhibit preadipocyte autophagy [162]. Celastrol can disrupt the link between endoplasmic reticulum stress (ERS) signaling pathways and downstream inflammation and lipid metabolism [163]. Thus, it inhibits ERS, inflammation, and adipogenesis, and promotes hepatic lipolysis to achieve effective weight loss.

In direct antitumor action, Celastrol can induce tumor cell apoptosis by downregulating the anti-apoptotic protein B-cell lymphoma 2 (Bcl-2) and upregulating the pro-apoptotic protein BCL2-associated X (Bax) [164]. It can also inhibit cell viability via the AMPK-mediated PLK-2 (Polo-like kinase 2, a serine/threonine-protein kinase) pathway to inhibit the growth of the human breast cancer cell line MCF-7 [165]. In addition, Celastrol displays anti-gastric cancer effects by targeting the antioxidant enzyme peroxiredoxin-2 in tumor cells to overexpress ROS and induce cancer apoptosis [166,167].

### 5.7. Curcumin

Curcumin, a phenolic active ingredient extracted from the rhizomes of Curcuma plants, shows significant potential in colorectal cancer treatment. Curcumin can inhibit the binding of NF-κB to IKK and the activation of the NF-κB-DNA complex, ultimately downregulating the gene expression of NF-κB-mediated adhesion molecules, such as VCAM-1 (vascular-cell adhesion molecule 1), ICAM-1 (intercellular adhesion molecule 1), and ELAM-1 (endothelial-leukocyte adhesion molecule 1), thereby inhibiting tumor metastasis [168]. However, the clinical translation of curcumin is limited by its low bioavailability [169,170].

### 5.8. Ursolic Acid

Ursolic acid is a triterpenoid component widely distributed in plants, with preventive and therapeutic effects against cancer. As demonstrated in the introductions of various drugs above, cancer and obesity share multiple molecular targets and signaling pathways, and the induction of both is closely linked to inflammation. Experimental studies show that ursolic acid can inhibit cancer cell growth. For instance, accumulating data show that it can inhibit cell proliferation and induce cancer cell apoptosis of many tumors, such as liver cancer (e.g., HepG2) [171], colorectal cancer (e.g., HT-29) [172], and breast cancer (e.g., MCF-7) [173].

Using natural products for managing metabolic disorders such as obesity and cancer is one of the treatment strategies. However, there are some challenges and translation barriers when using natural products as a therapeutic intervention. For example, complex formulation, diverse bioactivity, manufacturing procedure, and evaluation of therapeutic efficacy need to be well understood and analyzed to increase their targeted effects and reduce potential side effects. In addition, the delivery method for targeted therapy of bioactive natural compounds should also be considered for their application.

## 6. Conclusions

Obesity is a multifactorial chronic disease, with excessive fat accumulation in adipose tissues. Adipose tissues secrete a variety of adipokines, cytokines, and chemokines to alter immune cell responses and cause chronic metabolic disorders. In cancer patients, obesity contributes to the development of an immunosuppressive microenvironment by promoting cancer survival, proliferation, and metastasis and inducing immune cell exhaustion and dysfunction. Several key signaling pathways, such as PI3K/AKT/mTOR, NOTCH, and HIF-1α signaling pathways, are involved in obesity-associated cancer development and progression. Natural product medicines have multiple targets and functions, and display promising therapeutic potential to treat obesity-associated cancer and metabolic disorders.

There are many challenges and barriers in the management of obesity-related cancers. Complex biological processes influence the development and progression of obesity and cancer. Factors impacting the efficacy of cancer immunotherapy in patients with overweight/obesity are complicated. Pre-clinical studies and clinical studies have shown that obesity increases inflammation and cancer progression in most cases. Controversially, obesity also demonstrates a beneficial effect in improving anti-tumor immunotherapeutic efficacy in some cancers, such as renal cancer. This “obesity paradox” phenomenon increases the complex roles of obesity in different cancers. Understanding the mechanism of obesity impacting different cancers helps develop obesity-targeted cancer therapy. The evidence from clinical trials provides translational guidance in the treatment of overweight and obese patients with cancer. For example, regarding day-to-day clinical oncology management, proper interventions such as weight loss, dietary intervention, and personalized nutritional plans can be applied to facilitate weight control and improve the quality of life. Sufficient exercise and physical activity are helpful to maintain body structure and reduce fat accumulation. Precision medicine and personalized treatment will also be helpful in this situation. The roles of obesity in cancer development risk and therapy in patients can be evaluated by its roles in the recruitment of different immune cells, including cytotoxic CD8 T cells and immunosuppressive myeloid cells. Therefore, both pre-clinical and clinical research studies are required in the future to precisely dissect the underlying cellular and molecular mechanisms of obesity-related cancer progression or suppression in cell and animal models and clinical studies. In addition, new therapeutic targets or diagnostic biomarkers are needed to better manage and treat obesity-associated cancers.

As artificial intelligence (AI) rapidly becomes a transformative approach in healthcare, AI-driven approaches can be harnessed to improve the daily healthcare of patients, increase innovative research findings, and accelerate the process of personalized treatment [174,175]. Big databases from clinical samples in patients with obesity-related cancers and health controls worldwide, and information on treatment and diagnosis, pave the way for exploring novel diagnostic markers and evaluating treatment efficacy. Machine learning and AI-driven approaches are useful tools and methods to predict the risk of obesity in cancer development and progression, and evaluate the treatment efficacy [176]. They have been applied to facilitate the discovery of novel biomarkers and therapeutic targets. The methodology for targeted delivery can be further advanced based on the findings of machine learning and AI technologies. In drug development, such as screening molecules, toxicity prediction, and bioactivity assessment, AI-driven approaches have provided promising results in accelerating the drug discovery process and the translation from pre-clinical to clinical practice [177]. Furthermore, the AI-driven approach could assist clinical decision-making, and AI-driven technology could be applied for developing portable health tracking devices for daily healthcare monitoring.

## Figures and Tables

**Table 1:** Obesity impacts cancer development, treatment, and prognosis.

Cancers	Clinical Trials	Intervention	Functions	References
Differentiated thyroid cancer	Randomized controlled trial	Development	Patients with obesity have an increased risk of developing differentiated thyroid cancer (DTC).	[16]
Colorectal cancer	Canadian National Breast Screening Study	Development	Obesity (BMI ≥ 30 kg/m^2^) was associated with an approximately 2-fold increased risk of colorectal cancer among women who were premenopausal at baseline.	[17]
Acute lymphoblastic leukemia	NCT00558519 *	Prognosis	Pretreatment obesity (BMI ≥ 30 kg/m^2^) was significantly associated with worse overall survival rates of patients.	[18]
Colorectal cancer	Randomized controlled Trial	Prognosis	A higher BMI increased the risk of colorectal cancer mortality.	[19]
Breast cancer	NCT01140282	Treatment	A 16-week aerobic and resistance exercise improved physical fitness and quality of life in ethnically diverse overweight or obese breast cancer survivors.	[20]
Prostate cancer	NCT03261271	Treatment	A weight-loss intervention reduced obesity-related biomarkers of prostate cancer progression, such as insulin, cholesterol component, leptin/adiponectin ratio, visceral adipose tissue, C-reactive peptide, plasminogen-activator-inhibitor-1, and T cell count, as well as the quality of life of cancer patients.	[21]
Advanced refractory or late-stage solid tumors	NCT02743637	Treatment	Treatment with evexomostat (SDX-7320), a novel antiangiogenic and antimetastatic drug with insulin-sensitizing and anti-obesity properties, decreased plasma levels of low-density lipoprotein (LDL) cholesterol and leptin and insulin resistance, but increased plasma levels of adiponectin and high-density lipoprotein (HDL) in patients.	[22]

* Case number ID in the weblink of https://clinicaltrials.gov/.

## Data Availability

All the data supporting the reported findings can be found in this study.

## Abbreviations

The following abbreviations are used in this manuscript:

Acs-2	acyl-CoA synthetase
AGEs	Advanced glycation end products
AKT	Protein kinase B
AMPK	5′-adenosine monophosphate (AMP)-activated protein kinase
ATMs	Adipose tissue macrophages
BAP1	BRCA1-associated protein 1
Bax	BCL2-associated X
Bcl-2	B-cell lymphoma 2
BMI	Body mass index
BRCA1	Breast cancer susceptibility gene 1
C/EBPβ	CCAAT enhancer-binding protein β
CCK	Cholecystokinin
CCL	Chemokine (C-C motif) ligand
CCR	C-C chemokine receptor
CD8	Cluster of differentiation 8
c-Jun	Jun proto-oncogene
CRP	C-reactive protein
CTLA-4	Cytotoxic T-lymphocyte-associated protein 4
CVD	Cardiovascular diseases
CXCL	Chemokine (C-X-C motif) ligand
CXCR	Chemokine receptors
DTC	Differentiated thyroid cancer
EC	Endometrial cancer
EGFR	Epidermal growth factor receptor
ELAM-1	Endothelial-leukocyte adhesion molecule 1
EMT	Epithelial–mesenchymal transition
EOC	Epithelial ovarian cancer
ERK1/2	Extracellular signal-related kinases 1 and 2
ERS	Endoplasmic reticulum stress
FAT-6	Stearoyl-CoA desaturase
FFA	Free fatty acid
G-CSF	Granulocyte colony-stimulating factor
GM-CSF	Granulocyte-macrophage colony-stimulating factor
G-MDSC	Granulocytic myeloid-derived suppressor cells
GzmB	Granzyme B
HCC	Hepatocellular carcinoma
HDL	High-density lipoprotein
HepG2	Liver cancer cells
HIF1α	Hypoxia-inducible factor 1 alpha
ICAM-1	Intercellular adhesion molecule 1
IFN-γ	Interferon gamma
IKK	Inhibitory-κB kinase
IL-6	Interleukin 6
iNOS	Inducible nitric oxide synthase
IR	Insulin resistance
IRS-1	Insulin receptor substrate 1
JAK2	Janus kinase 2
JNK	c-Jun N-terminal kinases
Kat-1	3-ketoacyl-CoA thiolase 1
Ki-67	Marker of proliferation Kiel 67
KRAS	Kirsten rat sarcoma virus
Lag3	Lymphocyte activation gene 3 protein
LAMs	Lipid-associated macrophages
LD	Linear dichroism
LDL	Low-density lipoprotein
MAPK	Mitogen-activated protein kinase
MASLD	Metabolic dysfunction-associated steatotic liver disease
MCF-7	Michigan cancer foundation-7 cancer cell line
MCP-1	Monocyte chemoattractant protein-1
MDSC	Myeloid-derived suppressor cell
Mdt-15	Mediator subunit MDT-15
MHC-II	Major histocompatibility complex class II molecules
MIP-1α	Macrophage inflammatory protein (MIP)-1α
MIP-1β	Macrophage inflammatory protein (MIP)-1β
M-MDSCs	Monocytic myeloid-derived suppressor cells
mTOR	Mammalian target of rapamycin
mTORC	Mammalian target of rapamycin complex
MYC	Myelocytomatosis oncogene
NF-κB	Nuclear factor kappa-light-chain-enhancer of activated B cells
NK cell	Natural killer cell
NKT cell	Natural killer T cell
NLRP3	NLR family pyrin domain containing 3
NOTCH	Neurogenic locus notch homolog protein
NSCLC	Non-small cell lung cancer
p53	Tumor protein p53
PCNA	Proliferating cell nuclear antigen
PD-1	Programmed cell death protein 1
PD-L1	Programmed cell death ligand 1
PGC1α	Peroxisome proliferator-activated receptor gamma coactivator 1-α
PI3K	Phosphoinositide 3-kinases
PKC	Protein kinase C
PLK-2	Polo-like kinase 2
pod-2	Gene encoding acetyl-CoA carboxylase
PPARα	Peroxisome proliferator-activated receptor-alpha
PPAR-γ	Peroxisome proliferator-activated receptor γ
PRDM16	PR domain containing 16
RAB7	Ras-related protein
ROS	Reactive oxygen species
S6	Ribosomal protein S6
SETD2	SET domain-containing 2 protein
SHH	Sonic hedgehog protein
SLC7A5	Solute carrier family 7 member 5
SREBP1c	Sterol regulatory element binding protein 1c
SREBP2	Sterol regulatory element-binding protein 2
STAT3	Signal transducer and activator of transcription 3
STC-1 cells	Intestinal secretin tumor cell line
T2D	Type 2 diabetes
TGF-β	Transforming growth factor-β
TGR5	Takeda G protein-coupled receptor 5
TGs	Triglycerides
Th1	Type 1 T helper cell
Th17	T helper type 17
TILs	Tumor-infiltrating lymphocytes
Tim3	T-cell immunoglobulin and mucin domain 3
TLR4	Toll-like receptor 4
TNF-α	Tumor necrosis factor alpha
Treg	Regulatory T cell
Trem2	Triggering receptor expressed on myeloid cells 2
UCP1	Uncoupling protein 1
VAMP7	Vesicle-associated membrane protein 7
VAT	Visceral adipose tissue
VCAM-1	Vascular-cell adhesion molecule-1
VEGF	Vascular endothelial growth factor
Wnt	Wingless-type MMTV integration site family
ZEB1	Zinc finger E-box binding homeobox 1
